# On Smart Geometric Non-Destructive Evaluation: Inspection Methods, Overview, and Challenges

**DOI:** 10.3390/ma15207187

**Published:** 2022-10-15

**Authors:** Ali Jaber, Sasan Sattarpanah Karganroudi, Mohammad Saleh Meiabadi, Ahmad Aminzadeh, Hussein Ibrahim, Mehdi Adda, Hossein Taheri

**Affiliations:** 1 Institut Technologique de Maintenance Industrielle, 175, Rue de la Vérendrye, Sept-Îles, QC G4R 5B7, Canada; 2 Département de Mathématique, Informatique et Génie, Université du Québec à Rimouski, Rimouski, QC G56 3A1, Canada; 3 Department of Manufacturing Engineering, Équipe de Recherche en Intégration Cao-CAlcul, Université du Québec à Trois-Rivières, 555 Boul de l’Université, Drummondville, QC J2C 0R5, Canada; 4 Department of Mechanical Engineering, École de Technologie Supérieure, 1100 Notre Dame West, Montreal, QC H3C-1K3, Canada; 5 Department of Manufacturing Engineering, Georgia Southern University, 332 Southern Drive, Statesboro, GA 30458, USA

**Keywords:** non-destructive evaluation (NDE), artificial intelligence (AI), machine learning (ML), Industry 4.0, smart inspection

## Abstract

Inspection methods, also known as non-destructive evaluation (NDE), is a process for inspecting materials, products, and facilities to identify flaws, imperfections, and malfunctions without destruction or changing the integrity of materials, structures, and mechanisms. However, detecting those defects requires test conducting and results inferring, which is highly demanding in terms of analysis, performance, and time. New technologies are therefore needed to increase the efficiency, probability of detection, and interpretability of NDE methods to establish smart inspection. In this context, Artificial intelligence (AI), as a fundamental component of the Industry 4.0, is a well-suited tool to address downsides associated with the current NDE methods for analysis and interpretation of inspection results, where methods integrating AI into their inspection process become automated and are known as smart inspection methods. This article sheds a light on the conventional methods and the smart techniques used in defects detection. Subsequently, a comparison between the two notions is presented. Furthermore, it investigates opportunities for the integration of non-destructive evaluation (NDE) methods and Industry 4.0 technologies. In addition, the challenges hindering the progress of the domain are mentioned as the potential solutions. To this end, along with Industry 4.0 technologies, a virtual inspection system has been proposed to deploy smart inspection.

## 1. Introduction

Overall testing, inspection, and certification services have been continuously carried forward along with INDUSTRIAL revolutions. The testing, inspection, and certification market is projected to grow from $221 billion in 2022 to $268.9 billion by 2027 [1]. Testing, inspection, and certification services help manufacturers improve productivity, meet global standards, and enhance product and service quality for any product, service, or process. Part of that has been due to testing, inspection, and maintenance developments to keep up the production and service processes and ensure infrastructure health and safety regardless of the sector in which the company operates. Of all these developments, non-destructive evaluation methods (NDE) are of great importance because of their detection and characterization capabilities. Non-destructive evaluation is a set of techniques that do not destroy materials during inspection [2]. NDE is the evaluation and analysis technique that enables inspecting materials or components without causing any damage to the part [3] in a safe and reliable manner. Moreover, NDE methods are cost-effective and safe for workers. These methods are mainly used in aerospace and manufacturing industries [3] and vary from visual inspection performed by engineers specialized in the field to chemical and liquid penetration tests, alternative current, acoustics, etc. [4].

However, the contemporary industry is struggling with new challenges that prohibit modernization and synchronization. Due to the large volume of data and innovative technologies that are emerging exponentially and in response to the need for better and faster production, the world has entered a new stage of the industrial revolution, which is also called Industry 4.0. Industry 4.0, defined as the digital transformation of the business [5], is a relatively new concept that includes the most important technological developments in the fields of automation, control and information technology applied to production processes. It incorporates concepts ranging from physical-cyber systems to the Internet of Things (IoT) and Internet services, and production processes are becoming more efficient, autonomous and adjustable [6]. Driven by a large amount of available data [7], Industry 4.0 is transforming manufacturing into smarter automation in many ways, including the installation of intelligent robots, sensors, the use of collaborative robots, and the use of manufacturing simulations [8]. An Industry 4.0 typical example is the introduction of a monitoring system that monitors equipment in real-time by the means of AI algorithms to automatically detect and identify defects [9].

### Industry 4.0 and Quality Control

On the other hand, both machines and managers are regularly challenged with decisions, including massive data acquisition and customization in the production process. One of the primary issues in this area is to predict the requirements of assets for being in well-maintained condition at some point in the future. The ability to perform predictive maintenance helps improve machine downtime, costs, control, and production quality or what are known as “Quality Management” parameters [10]. Quality management, also termed as Quality Control (QC) refers to the procedures or inspections implemented during the manufacturing process to ensure product quality. In short, QC is the process of verifying that manufactured goods meet predetermined specified requirements.

**Figure 1:**
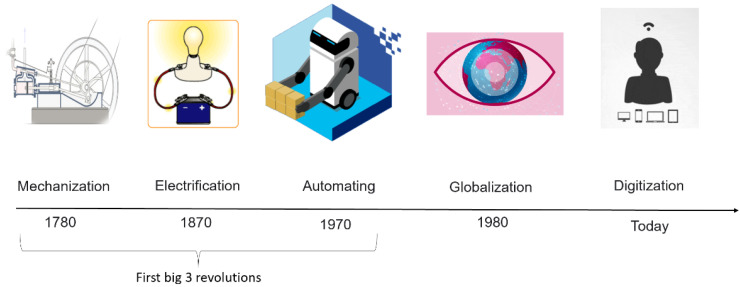
The different ages of industrial revolutions.

Additionally, the expansion in sensing technologies paves the way for the potential to capture 100% of measurement data and brings the 100% control concept into reality. This is a significant breakthrough in the digitization era as each of the defective items can now be identified and thus separated [11]. In this context, several implementations have been carried out where Industry 4.0, specifically smart inspections, has been used in QC management, as in [12,13,14]. Apart from directly improving QC, the Industry 4.0 toolset can enhance quality at all stages of the production process, such as the quality of information required for optimization, planning and operation, the quality of forecasting, simulation, and prototyping, which can even lead to better employee engagement and participation. Figure 1 depicts different ages of industrial revolutions over five decades.

Today, with the huge amount of data (big data) available [15] and the continuous development of digitization technology integrated into smart factories, a new type of inspection known as smart inspection has been introduced. In this article, Section 2 presents conventional NDE methods classified based on the notion of inspection. In addition, the advantages and drawbacks are mentioned. Next, Section 3 defines smart inspection tools used in defect detection, while Section 4 describes the different components of Industry 4.0 and lists several related applications. Section 4 also records challenges and future recommendations. Finally, Section 5 proposes a model using digital twins for the smart inspection of lattice towers. This article contributes to the academic knowledge according to the below:

- To the best of our knowledge, this is the first article to list the traditional inspection methods, discusses their advantages and disadvantages and compare them to the new smart techniques to act as a gathering point of all intertwined areas: artificial intelligence, Industry 4.0, inspection and maintenance;
- Listing the challenges hampering the domain and the relevant future recommendation in a manner that helps researchers to know where we stand today and where to start their new work;
- Proposing a new model that is based on digital twins for smart inspection of lattice towers.

## 2. Classification of NDE Methods

In general, NDE methods can be classified based on various factors, including the type of physical field, type of defect, and performance characteristics. In order to classify NDE methods based on physical fields, electric, magnetic, thermal, and mechanical fields can be highlighted. Defects can also be classified into surface and subsurface defects. Accuracy, efficiency, safety, and cost are the most important determinative parameters. Table 1 presents an overview of some of the NDE methods.

### 2.1. Visual Evaluation (VE)

Visual evaluation is the process of collecting data visually. This type of inspection requires a team of trained and experienced inspectors and usually does not require a special set of tools. It is effective to capture macroscopic defects, bad joints, incorrect dimensions, inadequate surface finish, large cracks, and non-compliant parts. It is also used to detect defects in composite structures [3]. Optical-based NDE techniques, as the major subset of VE methods, offer many advantages compared to other NDE methods. In addition to resistance to electromagnetic interference, optical NDE is not limited to a specific type of material and can be used in various cases. Although the use of optical approaches is expensive, these advantages lead to a wide range of applications [16]. That being said, the VE inspection methods cannot be limited in number, where any technique using vision can be considered in the mentioned scope. However, robust and efficient implementations of VE can be obtained in the literature, such as the example provided in [17]. The authors in this article presented a vision measurement system that was designed to track the complete field deformations of specimens, and a quad ocular vision system to calculate the concrete column deformations in this study. In this context, the state-of-the art of the VE system in infrastructure and concrete can be studied in detail in article [18].

### 2.2. Eddy Current Evaluation

Eddy-current testing offers a high degree of sensitivity for material identification and microstructure characterization [19]. As the name suggests, eddy current evaluation is based on introducing an alternating current into a conductor through a process called electromagnetic induction [3]. The interaction between the magnetic field source and the test material induces eddy currents in the test piece [20]. Inspectors can detect the presence of very small cracks by monitoring changes in the eddy current. Eddy current allows detecting cracks in a variety of conductive materials, both ferromagnetic and non-ferromagnetic. It is applied without any contact between the test object and the sensor [21]. Eddy current inspection has proven its effectiveness in defect detection. Its implementations are seen in different domains such as the aeronautics industry [22,23], nuclear industry [24,25], metallurgical industry [26,27], and transportation [28].

### 2.3. Ultrasonic Evaluation

Ultrasonic evaluation is based on the propagation of ultrasonic waves into an object. These high-frequency sound waves are radiated into the material to characterize the material and detect defects. It consists of several functional units such as pulser-receivers, piezoelectric transducers, and display devices. A pulsar is an electronic device used to generate high voltage electrical pulses. Using pulses, the transducer generates high-frequency ultrasonic energy. Sound energy is introduced that propagates through the material like a wave. If there is a discontinuity in the wave path such as a crack, some of the energy will be reflected by the flat surface. The reflected wave signal is converted into an electrical signal using a piezoelectric transducer and the output is displayed on the screen [29]. Surface cracks can also be detected using ultrasonic evaluation. It uses the same concept as naval SONAR. In ultrasonic evaluation, information about the ultrasonic wave, such as its reflection and scattering, is used to detect flaws in materials. The two most common forms of sound waves used in industrial inspections are the longitudinal wave and the shear wave. The traveling speed of shear waves is almost half of longitudinal waves. It was found that Rayleigh waves, which are one of the surface waves that are travelling along the solid surface, are more suitable for surface flaw detection due to their physical properties [30]. In literature, there are several implementations of ultrasonic inspection. In [31], for example, the authors suggested a system model for ultrasonic examination of smooth planar fractures in ferritic steel employing pulse-echo probes. Their suggested model predicts echo amplitudes and ranges as functions of probe location, and is implemented as a suite of adaptable and user-friendly computer programs suited for usage by practical NDE engineers, backed up by a thorough user manual. Similarly, in [32], the authors introduced Laser-EMAT (ElectroMagnetic Acoustic Transducer), an ultrasonics technology appropriate for on-line surface and interior fault detection in a steel mill. The device is designed to autonomously check steel as it travels through the steel production process at temperatures above 700 degrees Celsius. Because of its non-contact nature, it is one of the few ultrasonic systems that could ever be employed in the rigorous working environment of a steel mill.

### 2.4. Thermal Inspection

Thermal inspection is a method of mapping and measuring surface temperatures. It is known for its use of thermal measurements of an object and its response to a stimulus. The most commonly used tools for temperature measurements are thermal cameras. Thermal cameras have been used to diagnose electrical junctions in power transmission networks [33], monitor the thermal state of other electrical installations automatically [34], assess particular qualities in various materials, and to assess the erosion resistance of silicon rubber composites as in [35]. It has proven to be an effective and economical method for evaluating concrete [3]. Thermal non-destructive evaluation is most likely applied due to its underlying physical principles. Infrared/thermal, X-ray, electromagnetic, and ultrasonic tests use the injection of some form of energy and detect the residual energy not absorbed by the object. Defects are detected when the energy intensity varies because of the presence of defects. Its results can be affected by thermal noise, but with sufficient combination with other NDE methods, more efficient and productive results can be achieved [36].

### 2.5. Laser Spot Thermography

Laser spot thermography (LST) is a new infrared evaluation method for surface crack inspection. The LST method uses a laser to produce a highly localized heating spot near the crack and an infrared camera to detect the perturbation of the round lateral heat flow to reveal crack information. Fiber-guided LST systems have been developed to inspect surface cracks in metal structures. A high-power laser, a fiber delivery unit, and a specially designed optical head are used to generate a heat source for the nine-by-nine laser array points on the sample surface. Combined with an improved image processing method that uses multiple background-free images, this system was able to successfully detect and extract cracks from measured thermal images [37].

## 3. Smart Inspection

Defect detection by traditional inspection methods is hindered by poor real-time capabilities, low inspection reliability, worker safety, and excessive costs that do not meet industrial requirements [38]. There are also several disadvantages to manual inspection. First, the yield of human beings decreases over time, so long-term work affects the inspector’s performance. In addition, training a pool of experienced and qualified inspectors is time-consuming, so it has low scalability. This leads to a slower inspection process which limits the throughput of the production line [39]. Based on what was presented earlier, the smart inspection can be principally defined as an inspection process to detect defects or anomalies without human intervention. Smart inspection is aimed to increase the reliability and safety of the products, equipment, and operations while keeping profitability and persistence in industrial services. Smart inspection has the ability to continue tracking the maintenance history of every piece of a facility with the aid of technological advancements offered by artificial intelligence techniques.

### 3.1. Artificial Intelligence (AI)

Artificial intelligence (AI) is the science and engineering of building intelligent machines, especially intelligent computer programs. This relates to the similar task of using computers to understand human intelligence, but AI does not have to limit itself to methods that are biologically observable [40]. Alan Turing, also known as the father of computer science, asked the question: “Can machines think?”, which led to the famous test known as the Turing test, in which an examiner tries to guess which of the participants is human and which is a machine based on the responses [41]. Stuart Russell and Peter Norvig discuss four definitions of artificial intelligence that separate computer systems based on rationality and action [42].

### 3.2. Machine Learning (ML)

**Figure 2:**
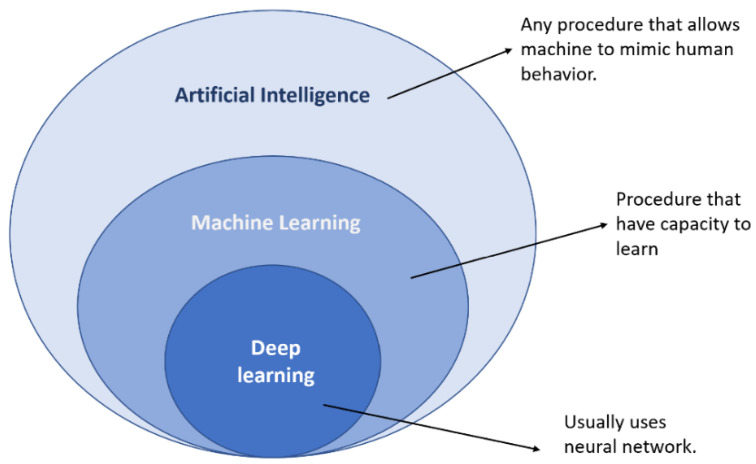
Machine learning and artificial intelligence concepts.

Machine learning (ML) is a subfield of computer science that focuses on methods and techniques for automating solutions to complex problems that are difficult to address using standard programming methods. Figure 2 shows that machine learning is a subset of artificial intelligence [43].

#### 3.2.1. Supervised Learning

Supervised learning is a machine learning technique that differentiates using labeled data sets. These datasets are intended to train or supervise algorithms in correctly identifying data or predicting outcomes. The model can learn and improve its accuracy over time using labeled inputs and outputs. An algorithm is used in classification to correctly allocate test data to specific groups. Regression is another form of supervised learning approach that uses an algorithm to identify the relationship between dependent and independent variables. Regression models are useful for predicting numerical values based on various data points, such as sales revenue projections for a particular business.

- Support Vector Machine (SVM): SVMs are supervised learning models with learning algorithms for data classification and regression analysis. SVM has the ability to manage multivariate datasets with high dimensions [7];
- Back Propagation Neural Network (BPNN): Back Propagation Neural Network (BPNN) is a supervised learning approach that uses iterative optimization to deal with classification or regression problems. It usually accepts a vector as input and returns a label containing information about the corresponding classes or function value.

#### 3.2.2. Unsupervised Learning

Unsupervised learning is defined as clustering and analyzing machine learning techniques on unlabeled datasets. These algorithms identify hidden patterns in data without the need for human interaction (hence the term unsupervised). Unsupervised learning models perform three main tasks: clustering, association, and dimensionality reduction.

- Artificial Neural Network (ANN): ANN consists of a network of interconnected units or nodes known as artificial neurons. An artificial neural network is in fact constructed from simulated neurons. Each neuron is a node that is connected to other nodes by connections. Each link has a weight that indicates the intensity of influence of one node on another node;
- Convolutional Neural Network (CNN): CNN is a class of ANN that is mostly used for analyzing visual images. A CNN consists of three layers: an input layer, hidden layers, and an output layer;
- Region Based Convolutional Neural Networks (RCNN): R-CNN or RCNN is a type of machine learning model used for computer vision tasks, especially object recognition. These networks are slow in identifying areas and scanning. RCNN runs CNNs on each region and the output is fed to and SVM for region classification;
- Fast- RCNN: Fast R-CNN uses a less complex architecture than RCNN. It is faster in identifying regions and has improved performance. Fast R-CNN uses a CNN for the whole image because it extracts features before selecting regions;
- K-Nearest Neighbor algorithms: The K-Nearest Neighbor (KNN) approach assumes that comparable data points in a dataset will be spatially adjacent to each other or neighbors. Although KNN is classified as a supervised learning method, it can solve both regression and classification problems. The KNN algorithm considers point proximity as a metric of class similarity;
- K-Means Algorithm: The K-means method divides the data into k clusters, each of which is characterized by a centroid. A cluster is a collection of data points that are grouped in space due to having similar characteristics. The centroid is also where the center of the cluster is located;
- Autoencoders: An artificial neural network known as an autoencoder is used to learn to efficiently encode unlabeled inputs (unsupervised learning). The encoding is checked and improved by trying to recreate the input from the encoding;
- Decision Tree DT: A form of supervised machine learning (in which you describe what the input is and what the associated outputs are in the training data) where the data is continuously separated based on a certain parameter. Two entities may be used to describe the tree: decision nodes and leaves. Decisions or consequences are represented by leaves, and the data is separated into decision nodes;
- Yolo Algorithm: “YOLO” is the abbreviation for “You Only Look Once”. This is an algorithm that finds and identifies different objects in a picture (in real-time). To detect objects in real time, the YOLO algorithm employs convolutional neural networks (CNN), requiring just one forward propagation through a neural network, as the name implies. This indicates that the complete image is predicted in a single algorithm run. The CNN is used to predict several class probabilities and bounding boxes at the same time. As an example of the use of the YOLO algorithm, a model based on the YOLOv4-tiny-algorithm was developed in [44] to detect and position the Camellia oleifera fruit. Despite variations in light, the algorithm demonstrated great positioning stability and resilient operation. The YOLO-Oleifera model detected each fruit picture in an average of 31 milliseconds, which is fast enough to fulfil the requirement for real-time detection.

#### 3.2.3. Machine Learning Tools

- TENSORFLOW: Free and open source software library for machine learning and artificial intelligence [45];
- Keras: A neural network application programming interface (API) for Python that is integrated with TensorFlow and used to generate machine learning models. Keras models provide a straightforward and user-friendly approach to building a neural network that TensorFlow can subsequently build. Keras runs on top of TensorFlow [45].
- PyTorch: An open-source machine learning framework based on the Torch library used for applications such as computer vision and natural language processing, primarily developed by Facebook’s AI research lab. This is open-source software distributed under the Modified BSD license [45].

### 3.3. Automated Inspection

The scientific development of NDE of materials has its basis in the interdisciplinary integration of a variety of different and complementary scientific and engineering methods. The development of inspection systems require additional handling technology and robotics, electronic hardware, computer science, and software, as well as mathematical algorithms in the numerical simulation. Automated inspection is an innovative solution for inspection systems including different phases of data acquisition, data analysis, data fusion, advanced robotics, and computer aided inspection. The application of robots and scanning NDE devices for automated data-acquisition in the inspection system based on options for data fusion are new innovations allowing automated inspection. NDE system robots are responsible for delivering the inspection instruments to the inspection target point, plus providing position coordinates for the NDE system to associate with the NDE data. The functional system components include the robot, controller, and NDE instruments, plus a computer or computers to fuse the data [46].

Common NDE techniques for robotic inspection applications include:

- UT techniques (single element and transmit-receive, phased array, immersion)
- Electromagnetic techniques
- VT techniques

**Figure 3:**
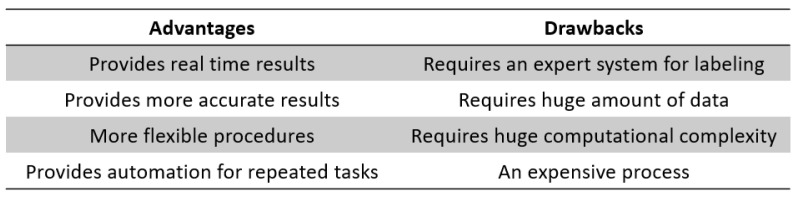
AI in smart inspection; advantages vs. drawbacks.

Data acquisition in multisensory systems has become an important approach for various research and application areas, where mainly non-invasive and non-destructive investigations are required. The data analysis of multisensory measurement systems offers several possibilities depending on the type and quality of collected data. An expert system is required to correlate the different analysis results and perform the assessment. Data fusion is generally defined as the use of techniques that combine data from multiple sources and gather that information in order to achieve inferences, which will be more efficient and potentially more accurate than if they were achieved by means of a single source [47]. More NDE applications in real time are now possible and within this context, automation is an important driver to integrate NDE into quality management and lifecycle management procedures. Automated inspection systems can be applied to diverse inspection technologies and to various types of non-destructive evaluation (NDE) techniques in order to increase reliability, data repeatability, flexibility, and speed. Figure 3 summarizes the advantages and drawbacks of using AI in smart inspection.

## 4. Industry 4.0

Industry 4.0 is the cyber-physical based revolution to connect the industrial world and digital world in order to exchange knowledge from the physical and virtual worlds [48] using automated information flow and data exchange regardless of location, time zone, or platform dealing with issues such as big data, smart factory, internet of things, digital twins, and smart inspection.

### 4.1. Big Data

By 2003, humans had generated five exabytes (1018 bytes) of data; however, this amount of data is now generated in two days. Big data refers to large data sets with large, diverse, and complex structures that are challenging to store, analyze, and visualize for subsequent processes or results. Big data is truly massive when it is diverse. Big data comes from various sources and is divided into three categories: structured, semi-structured, and unstructured data. Structured data is pre-labeled and easily sorted when it enters the data warehouse, but unstructured data is random and difficult to examine. Semi-structured data does not have defined fields and instead uses tags to identify pieces of data. Big data analysis refers to the technique of investigating huge volumes of data to discover hidden patterns and hidden relationships. This valuable information for businesses or organizations may assist them to gain richer and deeper insights and gain a competitive advantage. Consequently, big data initiatives must be carefully planned and implemented [49]. In addition, the volume of information has increased and the analysis of data sets has become more competitive. The problem is not only collecting and managing huge amounts of data of different types but also extracting meaningful results from it [50]. The integration of big data may affect a variety of industries, including entertainment, finance, healthcare, communications, security, manufacturing, and, of course, monitoring and inspection. The idea of big data creates the potential for the industry to create better value-added products. The goal is to obtain more value from the variety and volume of data with the ability to review and process them in real-time [51].

### 4.2. Cyber-Physical System

Cyber-Physical System (CPS) is a concept that combines the physical and digital worlds with analytical tools to increase industry efficiency. The widespread deployment of CPS is directly tied to Industry 4.0. Cloud computing, Internet of Things (IoT), smart technologies, and big data are examples of strategic technical concepts included in CPS. CPS serves as the foundation for a number of new technology areas, including but not limited to smart factory, smart manufacturing, smart cities, smart buildings, smart electric vehicles, mobile systems, wearable gadgets, and unique military systems [51,52].

### 4.3. Smart Factory

**Figure 4:**
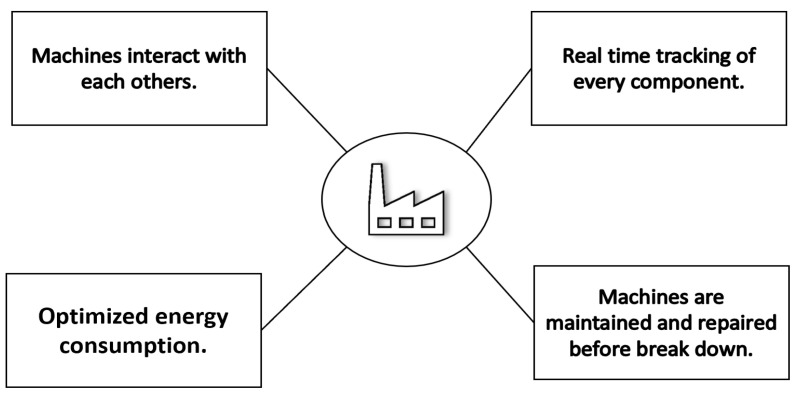
Smart factory features.

A smart factory is described as a manufacturing system that conveys an adaptive and flexible manufacturing process that is able to overcome new issues in a rapidly changing world of increasing complexity. Such a unique solution may be related to the growing automation, which is considered an integration of hardware, software, and/or mechanics that leads to optimization in the industrial unit. This method may result in less unnecessary work and less waste. In contrast, it may be considered from the perspective of cooperation between various industrial and non-industrial partners, where the smart component contributes to the construction of a dynamic organization [51,52]. Figure 4 represents the main characteristics of a smart factory.

### 4.4. Internet of Things

Internet of things (IoT) is a technology for connecting, updating, and adopting a network of smart devices according to changes in the operational condition. The network may identify physical items and digital entities indisputably using wireless mobile devices and integrated/standardized electronic identification systems. It allows the user to retrieve, store, transfer, and analyze data while maintaining the connection between the virtual and real worlds. However, from a more practical point of view, the Internet of Things means the standard and direct digital identification of a physical device (for example, through SMTP protocols, IP addresses, and HTTP, among others) through the use of a wireless network [51,52].

### 4.5. Augmented Reality

Augmented reality (AR) is considered one of the key technologies in Industrial 4.0. It creates the opportunity to look at the physical world through virtual objects combined with the physical world. AR technology usually has the following three characteristics: combining real and virtual worlds, real-time interaction, and being registered in 3D space. It also has at least six classes of potential applications, including medical visualization, entertainment, manufacturing and maintenance, process planning, and military aircraft navigation and targeting [53].

Based on current technology, the challenges of using AR in smart manufacturing can be summarized in four aspects as follows:

- Real-time reflection/real-time data: There is a huge amount of real-time data transfer during the manufacturing process. The transfer, analysis, and use of this data will be very important;
- 3D space registration: Rreal manufacturing environments are very complex. The perfection of detection, tracking, and following of target objects determines the quality of the AR;
- Reliability: In some extreme manufacturing environments, such as high temperature, low pressure and humidity conditions, the AR must be reliable and robust enough to perform the tasks;
- Collaboration: The use of AR in smart manufacturing must consider multiple users and operators simultaneously monitoring and controlling the target. Collaboration functionality is critical to AR applications.

By using augmented reality (AR), it is possible to display overlayed visualizations on the real object, improving the visualization and interpretation capabilities through physical and virtual context interactions. AR can transfer real time information to examiners while the NDE process is carried out and AR vision is illustrated at the same time in order to to enable a collaborative analysis. While augmented reality is mostly developed based on the real world, virtual reality (VR) relies on the virtual world. Virtual reality allows us to visualize a simplified representation of the part with all data transferred in real time. 3D visualization of real time NDE data should be automatically overlayed on digital representation of evaluated parts. Therefore, VR vision displays a combined illustration of NDE data and digital representation to enhance efficiency and reliability of analysis and interpretation. VR can be used in a digital twins system to compare physical and VR versions to provide detailed overviews and analysis.

### 4.6. Digital Twins

A digital twin (DT) is defined as a virtual representation of a physical system or process enabled through data and simulators for real-time prediction, optimization, monitoring, control, and improved decision making. So, a live digital representation (model) of a system can be developed which involves the creation and organization of digital elements of physical and functional characteristics [54]. The physical and virtual worlds are bridged through cloud networks with the aim of providing a comprehensive and reliable representation of a physical system. There are, however, challenges with ensuring the reliability of the digital twin. Digital twins are subjected to various kinds of uncertainties that can enter experimental measurements and mathematical models in various ways. Uncertainties arise from variations in material properties, process setting, but also from insufficient accuracy of the numerical models as a result of numerical errors or unrealistic assumptions. A crucial issue for quantification of uncertainty is how uncertainties in model inputs are propagated to uncertainties in model outputs. The obvious advantage of a reliable digital twin is that it can continuously adapt to operational conditions by monitoring, collecting and processing sensory data feeding into the digital twin in real-time. The realization of DT technology is certainly dependent on IoT technology. Digital twin synchronizes the state of a physical system with its digital representation using various internet of things (IoT) sensors and actuators. Continuous and online communication between different physical and virtual entities is only possible through the capabilities of the IoT to transmit large volumes of data. Digital twins can predict the future of their physical counterparts [55] and thus become an essential component of proactive and predictive maintenance strategies [56]. Digital twins should be able to deal with three levels of data: long-term, short-term, and real-time. A problem that may arise when dealing with the huge amount of data available is how to process the data in real-time or how to deal with big data.

**Figure 5:**
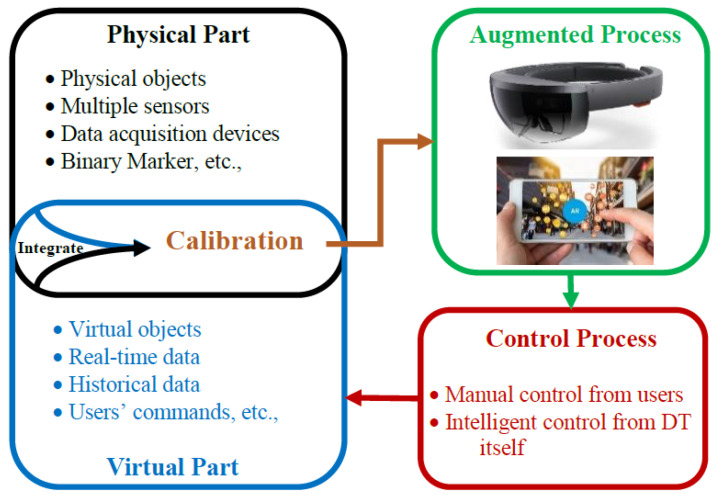
The framework of visualizing the digital twin data by using AR [53].

Augmented reality allows the users to visualize and interact with digital twin data at a new level that gives the opportunity to provide intuitive and continual visualization of digital twin data. The augmented digital twin data can provide more useful and comprehensive information to users through the AR device. Figure 5 shows the framework of visualizing the digital twin data by using AR. In order to achieve intuitive and flawless AR visualization of a digital twin, the 3D models in virtual part need to be perfectly aligned with the physical part, which can be obtained by the calibration process to accurately integrate these two parts [53].

### 4.7. Industry 4.0 for Monitoring and Inspection

This section presents examples of the implementation of Industry 4.0 technologies for monitoring and inspection. For bridge inspection, several models based on ANN are investigated [57]. These models were implemented to assist in the bridge inspection process. Each type of bridge has its own indicators and deterioration rates, and the focus will be on concrete beam bridges. Three algorithms—ANN, SVM, and DT—were used to develop twenty-seven prediction models for the failure state of concrete beam bridges. To evaluate the performance of the prediction model, four indices of accuracy, precision, recall, and F-score were selected. After reviewing and comparing the results, it was concluded that DT and ANN had better performance compared to the low performance of SVM. It was also concluded that ANN is widely used when it comes to predictions in infrastructure, but DT is more promising in terms of speed [58]. Microwave NDE was used to investigate the structural health of coated/uncoated metallic or dielectric materials. The aim was to use microwaves ranging from 8.2 GHz to 12.4 GHz to search for fractures in materials. The microwave sensor data was cleaned using feature extraction. This strategy helped to remove redundant data from the dataset and improve the learning accuracy for the most relevant attributes. When a fracture in the material was encountered, the waves were shifted horizontally to the left. With this information in mind, feature selection algorithms were used to pick the top five features that can be used to accurately detect fractures in materials. The characteristics are then used as data points by the KNN algorithm to automate the crack detection method. The accuracy of crack detection by the KNN method was 99.64% [59]. In order to improve the inspection process, the use of unmanned automated vehicle (UAV) was suggested to obtain more valuable data. Manually reviewing data is also a challenging task to perform. Computer Vision methods are used to automate the process of examining large volumes of available data. Edge detection is one of the conventional methods to detect cracked surfaces. A CNN-based model was generated using RESNET. Also, using a UAV, a method for monopole tower inspection, was proposed using a deep learning algorithm with the help of the TensorFlow framework, which enables automatic feature extraction [60]. Threshold-based crack segmentation, crack edge detection, and nonlinear filters to extract local crack information are methods that do not require manual configuration. A deep convolutional neural network (DCNN) model was developed consisting of six convolution layers, three fully connected layers, and two pooling layers. It should be noted that the convolutional layer is used to extract features. The pooling layer is used to reduce the dimensions of the features using the down-sampling function. There are two pooling approaches: average pooling and maximum pooling, and it was shown that maximum pooling is better when processing the CNN configuration. The fully connected layer is at the end of the DCNN where every neuron is fully connected to every neuron in the previous layer. Enhanced Chicken Swarming Algorithm (ECSA) was used to solve the meta-parameter optimization problem. This has led to an increase in the accuracy and convergence rate of the model. The combination between ECSA and DCNN performed better compared to other networks such as Alex Net, DenseNet-201 and others [61].

**Figure 6:**
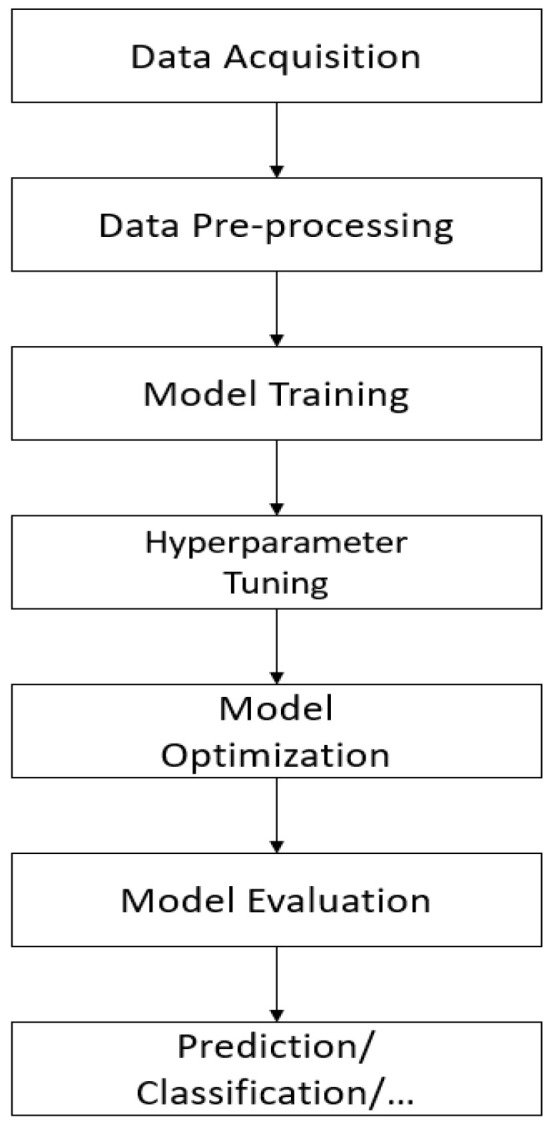
AI smart model workflow.

Several AI methods also suffer from long learning time, larger computational requirements, detecting certain defects, and low accuracy results. It was proposed to use the wavelet transform (to smooth the images and remove noise) to reduce the computation time, and the spectral measurement method was used to extract the features. Comparison between SVM model and BPNN showed that SVM model performs better than BPNN model [38]. Although a large image can be divided into small pieces for classification, it is difficult to decide the size of the small pieces, especially for geometrically complex products. Due to the lack of surrounding information, a small amount of background intermingled in the image may be detected as an error if the size is too small. If the size is too large, it may contain various sorts of faults that are difficult to spot in the image [39]. Predictive maintenance is also a hot topic in the world of Industry 4.0 and smart inspection. Updating maintenance technology within the framework of Industry 4.0 enables predictive maintenance, leading to a reduction in total downtime. Researchers have shown a strong interest in predicting the remaining useful life (RUL) of a machine and several prognostic algorithms were developed to do so. A prognostic algorithm predicts that a machine or a system or a component will stop doing what it was intended to achieve. In data-driven prognostic algorithms, several machine learning and deep learning algorithms are used to extract features and predict RUL with little human interaction [62]. Image processing is widely used when applying NDE, but automated approaches are rarely used. Two neural network-based classifiers were proposed for machine fault detection: a direct spot classifier and an indirect spot classifier. The direct spot classifier consists of a hidden layer (150 neurons), an input layer with 60 × 60 neurons (60 × 60 white and black images) and an output layer representing three classes (healthy, crack, and linear). The indirect classifier extracts some features after analysis (an additional step required for the indirect spot classifier). These features are used as input to the neural network, which will have a hidden layer (10 neurons) and an output layer (3 neurons). According to the results, it was concluded that the indirect classifier performs better than the direct classifier [63]. Figure 6 displays the common workflow to implement an AI smart model for automated inspection.

### 4.8. Challenges and Future Recommendations

Despite AI and Big Data’s exceptional performance, various difficulties lie in the way of its successful application in Industry 4.0. Several significant data-related obstacles, explainability and interpretation concerns, and adversarial and other security threats on AI in Industry 4.0 are mentioned below [64,65,66,67,68,69]:

- Data Concerns (availability—readiness—locality of data)
  ▪ Availability: Training a model necessitates having the relevant data, which may not be available at the time, or may be available but inaccessible for a variety of reasons;
  ▪ Readiness: Even if data is available and accessible, several issues should be taken into consideration such as data heterogeneity (the accessible data may have various attributes or be formed of various types), noise (Data may be contaminated by noisy qualities as a result of interactions between data collecting instruments and other electrical devices, affecting the overall outcomes of the ML models), and missing data;
  ▪ Data locality: In the actual world, data is dispersed into disparate and unrelated entities known as “Data Islands.” Data relating to the same issue and available on separate data islands cannot be accessed to be used and analyzed due to different policies and legislation.
- ML Model Concerns
  ▪ Accuracy and performance: Obtaining the maximum accuracy for ML models remains the primary aim for academics from many areas, with the best accuracy leading to the most adoption and integration of this technology;
  ▪ Explainability: Some ML models, particularly Deep Learning models, are identified by their black-box identification. Even when great accuracies are established, the lack of a how-it-worked explanation may reduce trust in those models;
  ▪ Model Selection: Even when working with the same problems, various models may provide different solutions. Support Vector Machines (SVM) and Logistic Regression (LR); for example, might provide different outcomes while working with the same data at the same time. As a result, finding the best model and fine-tuning its parameters is a difficult undertaking;
  ▪ Execution time and complexity: Because of the complexity of the data or models, the numerous preparation stages, and a variety of other factors, ML models may need massive computational resources and a lengthy execution time.
- Privacy:
  ▪ Privacy is one of the most pressing topics in the field of machine learning. Users may refuse to provide their data for a variety of reasons, affecting data availability and jeopardizing the entire ML cycle.

Following that, several future trends can be defined to overcome the mentioned challenges. Below are some of those recommendations:

- Data related recommendation: Before processing the information, extrinsic and intrinsic signal artefacts that obfuscate the signals should be eliminated or reduced. Several implementations, such as those listed in [70,71,72], have previously been built for this purpose.
- Model related recommendation: Smart inspection is becoming increasingly vital. Therefore, there is a need to enhance the accuracy of defect detection by the means of Industry 4.0, as well as the explainability of these tools, and to minimize as much as possible the black box features of the models incorporated in these smart techniques. Increased accuracy and explainability will help these devices gain confidence and, as a result, be deployed.
- Privacy recommendation: Later machine learning algorithms provide further privacy options. Federated learning (FL), for example, is a promising technology that can aid in the resolution of privacy issues. Federated learning is a sort of collaborative distributed/decentralized machine learning privacy-preserving solution that trains a model without transferring data from edge devices to a central server. Instead, the trained models are distributed across the edge devices and the central server, which serves as an aggregation station to generate the global model without knowing the embedded data [73,74,75,76,77]. The use of FL in defect detection and monitoring is predicted to assist overcome the privacy issue, allowing for greater data gathering and therefore improving accuracy.

## 5. Towards Smart Inspection for Industry 4.0

**Figure 7:**
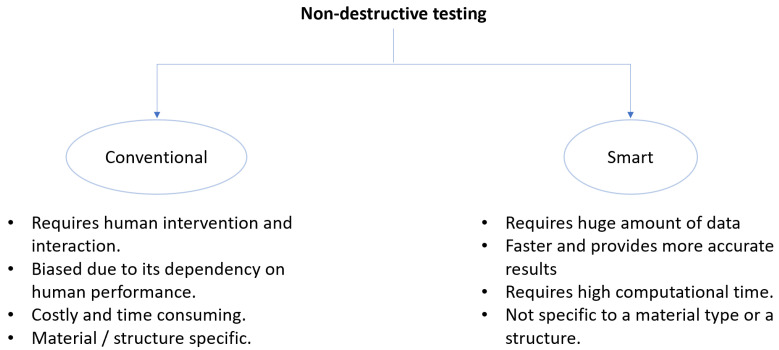
A comparison of conventional and smart inspection using nondestructive testing techniques.

Smart inspection is responsible for specifying, developing, operating and maintaining an inspection system and providing support during the preparation and operational phases of the inspection process without human intervention. Figure 7 compares conventional inspection with a smart inspection.

Smart inspection requires a huge amount of data, high computational time, and provides more accurate results without specific requirements for material and structure. With the advent of Industry 4.0, various industries are now considering a range of digital technologies to set up smart inspection. The integration of digital twins (DT) and inspection technologies makes the inspection process smarter and more sustainable.

### 5.1. Smart Inspection by Digital Twins

In this section, an initiative is proposed to establish smart inspection using the digital twins system as a contributor to innovation in inspection technologies. A smart inspection system can be established by a digital twins framework created from raw data (already available), previously collected data, and additionally acquired data during the process. Digital twins currently can offer a wide range of applications not only in the fields of operation and maintenance but also in the fields of monitoring and inspection. Digital twins are associated with a machine learning platform structured by collected data and integrated with numerical modeling as a baseline for further simulations to assess and estimate the state of a physical system [78]. Having built the digital twin, the virtual counterpart can be subject to evaluation tests in order to identify anomalies in the physical features in terms of material, design, assembly, and manufacturing. The digital twins demonstrate and quantify performance indications of the physical entity in a virtual evaluation using advanced numerical models in combination with detailed assessment data. A continuously maintained flow of data between the physical system and its twin allows evaluation and simulating of the properties of the physical system.

A digital twin might be set up using multi-scale data (from remote sensing, IoT sensors, and management systems) and numerical-based models in a common platform to train an intelligent data-driven model to predict, in advance or real-time, the occurrence and location of faults and defects. Deploying a digital twin for smart inspection dealing with data-driven operations requires:

- Machine learning and data-driven modeling
- A numerical modeling analysis
- Data-driven condition monitoring
- A real-time anomaly detection algorithm

In this context, digital twins have proven its success in the smart inspection field. Several implementations have been carried out in different research domains. For example, digital twins have widely been used in transportation sections such as tunnels, bridges, railways, and highways [79]. Besides, other applications belong to the domain of smart vision inspection, as the implementation mentioned and explained in [80]. Last but not least, digital twins are massively deployed in the civil engineering sector [81]. Some of those examples are the techniques proposed in [82,83]. In [82], authors developed a systematic technique for detecting and characterizing facade deterioration, locating as-built deviations from the design, and discovering clashes between structures. Moreover, authors of [83] managed to inspect defects by introducing six feature-based matching algorithms to match the components modelled in the Building Information Model (BIM) to the real world parameters, therefore detecting defects.

**Figure 8:**
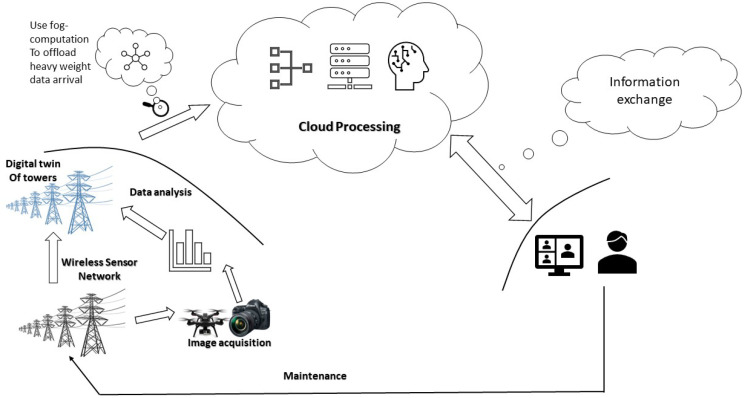
A model using digital twins for smart inspection of lattice towers.

Figure 8 depicts a data-driven monitoring system of lattice towers using digital twins for real-time anomaly detection. Such digital twins can be used for the smart inspection of lattice towers, enablingthe decision-making process. Based on maintenance protocols, data-driven modeling and analysis which are implemented onto the cloud network, appropriate decisions are made to efficiently detect the defects and alert the changes. One of the proposed solutions to offload heavy data arrival is to use fog computing. The digital twin of the lattice towers is updated using fog computation to deliver smart and sustainable inspection. This computing architecture is based on processing as much as possible before sending it to the data center. In this way, the heavy work from the central server to the fog nodes is offloaded and thus earns the ability to process very large data [15].

### 5.2. Virtual Inspection

Smart inspection involves the orchestration of traditional inspection techniques with cutting-edge Industry 4.0 technologies using artificial intelligence, machine learning, data analytics, and large-scale simulations that will drive digital twins for virtual inspection. Digital twins provide an opportunity to run both non-destructive and destructive evaluations within the digital platform on a virtual model. This type of evaluation could be called virtual inspection, which is feasible with the aid of a digital twin of a physical system. Virtual inspection may be used as a part of NDE examination in the inspection plan, as a substitute for physical evaluation, or as an additional quality control stage [84,85,86,87]. Virtual inspection is used to conduct examination and evaluation scenarios and is supposed to cover physical inspection shortcomings. Virtual inspection can be characterized by a fully integrated digital system supported by machine learning technologies and numerical modeling methods to measure the defects and locate the damages. Virtual evaluation can be used to control the quality of products and monitor the health of structures that are difficult to measure and/or access.

To perform a virtual evaluation, virtual models of a physical system are needed to represent the properties of physical systems, including physical and mechanical characteristics. These physical and mechanical characteristics are defined in the numerical model by parametric features describing the state of the system. Virtual scenarios can be planned for conducting virtual evaluations for observation of characteristic features. These virtual inspections also contain the portrayal of non-destructive evaluations on the virtual part. These virtual evaluations intend to simulate the actual behavior of the physical system corresponding to the experimental condition in real environments. Investigation of parametric features may assist to replicate physical responses of the physical system in virtual experimentations for non-destructive evaluations. The digital twin utilizes virtual models for evaluation simulations, as well as measured input and output data from the physical system in order to identically represent the system behavior. With the help of the digital twin, evaluation simulations can be performed for individual experiments and, based on the simulated data obtained, it can be determined which experiments are carried out on the physical system with high accuracy [88]. Therefore, the digital twin of a system will be able to cope with different sources of uncertainties, reduce the discrepancies between predicted results and the measured output data. The data obtained from a digital platform can also be of value in the design stage to obtain a vision of the physical system prior to production to determine optimized design properties to avoid plausible defects [89].

A virtual inspection involves designing fault detection and diagnostics algorithms to build a digital twin of a physical object to estimate the condition and predict inconsistency between measured data and the expected state. A virtual inspection system is capable of real-time monitoring of a physical object and real-time updating of a virtual model. The updated virtual model can be achieved by a continuous flow of new data from the measurement platform. Utilization of advanced visualization, imaging and analytics tools for measurement platforms for data acquisition and processing helps to increase the accuracy of the virtual inspection. An accurate digital representation of the critical area of a physical object can be created using dynamic information from real and simulated geometric data. A fully developed virtual inspection system is expected to

- Verify the accuracy of the digital twin;
- Diagnose if there is an inconsistency from the desired state;
- Figure out where the inconsistency originates;
- Adapt the digital twin by making appropriate changes.
- The final aim of the virtual inspection is to diagnose the assets’ health status with maximum reliability and to ensure requirements for accurate inspection of assets.

### 5.3. Image Classification

It is advised that image classification methods are robust techniques for anomaly detection systems. Computer vision and pattern recognition techniques have drawn great attention in non-contact inspection techniques to increase the objectivity, consistency and efficiency of the evaluation. Non-destructive detection of defects in thermal images of industrial materials based on segmentation of images was carried out using K-means clustering. K-means is an iterative technique that seeks to divide a data set into K separate non-overlapping subgroups (clusters) where each data point belongs to one and only one group. K-means was used for image segmentation [55]. There were three main parts that had to be divided. K is first initialized by selecting three distinct regions: the defect region; the area with increased thermal-wave accumulation as a result of containing a defect; and an area away from the defect. K-means was used alongside the canny edge detector, which is a multi-step algorithm used for edge detection. It consists of 5 steps that ends with edge detection. A 2D image resulting from image segmentation by K-means is fed to the detector. The results showed that the proposed method based on image segmentation can be successfully used to detect cracks.

An efficient support vector machine-based approach has been developed for automatic welding fault detection in real-time X-ray welding images, and cross-validation was used to find the best parameters of the SVM algorithm [56]. K-folder cross-validation is a popular cross-validation approach. The following are the basic phases of k-folder cross-validation: the data set is separated into k subsets, and the training and testing stages are performed k times. Each time, one of the k subsets is employed as the test set, while the remaining k − 1 subsets are combined to form a training set. Then, the average error of the total k trials is determined as the generalization error of the current model. The weld fault segmentation method is used for the extracted weld once it has been detected. 744 real-time X-ray weld images with 823 weld defects were obtained for testing in this research. The results demonstrate the effectiveness of the proposed method for fault detection.

It was proposed to develop an automatic detection system for defects of fasteners in railway tracks. The rail sleepers were located using the line segment detector and the fasteners were structurally modeled. Finally, the fastener objects’ Haar characteristics were utilized for failure detection [90]. Computer vision was used to identify the phenomenon of cable icing in transmission lines. The author introduced the Canny edge detection technique, which is based on support vector regression (SVR), and then integrated it with Hough and three gray-scale statistical features to identify icing irregularities. The SVR approach was used to predict the Canny parameters, and it was shown that the classification results based on this method outperform other methods [91]. A three-stage fault detection structure based on SSD and YOLO was developed for the contact network in high-speed railway lines. After locating the support device, the fasteners were discovered. Then, errors were identified using a deep neural network [92]. Physically checking for a missing nut and/or bolt on one of the world’s largest rail networks is extremely difficult, and as part of standard guidelines, rail inspections must be carried out every calendar month. This prompted the authors of [93] to develop an automatic detection system for missing nuts and/or bolts on the rail fishplate, thus reducing human errors. Images of a rail fishplate with different types of nuts and/or screws missing are used by the system. A similarity classifier trained with discrete wavelet transform DWT features is used to detect missing nuts and/or bolts on the rail fishplate. Next, the system reviewed a series of test photos to evaluate its accuracy. This program accurately detects the presence and absence of nuts and/or bolts on railroad fishplates. A high percentage of precision and recall was obtained, which indicates the excellent reliability and robustness of the proposed system.

In another study, a two-stage deep learning approach was proposed to inspect tiny missing fasteners. First, four classic data augmentation techniques are used to improve the minor defects of fasteners: rotation, noise, brightness and contrast shifts, clip, and an innovative defect augmentation method: copy-pasting. The ResNet-101 backbone is then used to extract defect features, which are then utilized to form a discriminative feature pyramid using lateral connectivity. In addition, an updated region proposal network (RPN) is used to generate defect suggestions. It optimizes the anchor size by varying the size length of the anchor based on the size of the defect region. Finally, a fully convolutional network (FCN) is used to construct pixel-wise segmentations on the defect bounding box, increasing the detection accuracy. This experiment investigated the faults of lack of pins, bolts, and rivets, and the findings confirmed the accuracy and high efficiency of the proposed method [94].

## 6. Conclusions

Improper maintenance of industrial facilities can have dire consequences. One recent addition to maintenance technology has been the advent of Industry 4.0 technologies for the implementation of proactive and predictive maintenance strategies. Industry 4.0 promises increased flexibility, mass customization, increased speed, improved quality, and increased productivity in manufacturing, allowing businesses to address various challenges such as increasingly individualized products, shorter lead time to market, and higher product quality. The age of smart inspection with the help of Industry 4.0 technologies has been emerging in recent years and will encompass almost everything within the maintenance industry.

In this paper, requirements to conduct a smart inspection that integrates traditional inspection technologies and Industry 4.0 technologies such as the Internet of Things (IoT), digital twins (DT), and machine learning (ML) techniques are described. Introducing these technological advancements in the field of maintenance and inspection increases reliability and guarantees the expected lifetime. Virtual inspection is associated with a smart inspection which is coordinated with Industry 4.0 technologies, in which we aim to develop self-learning digital twins to perform virtual evaluations.

## Figures and Tables

**Table 1:** An overview of the NDE methods and their advantages and disadvantages.

Methods		Visual Evaluation	Eddy Current	Ultrasonic	Thermographic Inspection	Radiographic Testing	Laser 3D Scanning	Laser Spot Thermography
	Criteria							
**Source**		-	Eddy current	Acoustic vibration	Thermal emissivity	X-ray/Gamma-ray	Laser beam	Heat distribution
**Material**		All	Conductive materials	All	All	All	All	Metals
**Contact requirements**		Non-contact	Non-contact	Contact/Non-contact	Non-contact	Non-contact	Non-contact	Non-contact
**Advantage**		Easy to implement, low cost	Low-cost, no surface treatment	Great depth penetration, high resolution	Full-field, fast, high resolution, high sensitivity	High resolution	Full-field, fast, high resolution	High surface temperature, hazardous environments, high resolution
**Disadvantage**		Surface defects, safety problems, time-consuming, low reliability	Scanner required	Sound attenuation, time-consuming, 2D measurements	Scanner required, detection of false positives, limitation due to thermal properties of materials	Radiation hazards, relatively slow, scanner required	Surface defects	Surface defects, time-consuming

## Data Availability

All data, material, and codes used in this paper are available.
